# Blood Urea Nitrogen-to-Albumin Ratio in Predicting Long-Term Mortality in Patients Following Coronary Artery Bypass Grafting: An Analysis of the MIMIC-III Database

**DOI:** 10.3389/fsurg.2022.801708

**Published:** 2022-02-18

**Authors:** Diming Zhao, Shanghao Chen, Yilin Liu, Zhenqiang Xu, Hechen Shen, Shijie Zhang, Yi Li, Haizhou Zhang, Chengwei Zou, Xiaochun Ma

**Affiliations:** ^1^Department of Cardiovascular Surgery, Shandong Provincial Hospital, Cheeloo College of Medicine, Shandong University, Jinan, China; ^2^Department of Ophthalmology, Cheeloo College of Medicine, Shandong Provincial Hospital, Shandong University, Jinan, China; ^3^Department of Cardiovascular Surgery, Shandong Provincial Hospital Affiliated to Shandong First Medical University, Jinan, China; ^4^Department of Cardiovascular Surgery, Shandong Provincial Hospital Affiliated to Shandong University, Jinan, China

**Keywords:** coronary artery bypass grafting, blood urea nitrogen, albumin, mortality, MIMIC III database

## Abstract

**Background:**

This study examined the role of blood urea nitrogen-to-albumin ratio (BAR) in predicting long-term mortality in patients undergoing coronary artery bypass grafting (CABG).

**Methods:**

In this retrospective cohort study, patients undergoing CABG were enrolled from the Medical Information Mart for Intensive Care III (MIMIC III) database. Patients were divided into the three groups according to the optimal cutoff values of BAR determined by X-tile software. The survival curve was constructed by the Kaplan–Meier method and multivariate Cox regression analysis was performed to explore the independent prognostic factors of 1- and 4-year mortality after CABG. The receiver operating characteristic (ROC) curves and the areas under the ROC curves (AUCs) were calculated to estimate the accuracy of BAR in predicting the outcomes. Subgroup analyses were also carried out.

**Results:**

A total of 1,462 patients at 4-year follow-up were included, of which 933, 293, and 236 patients were categorized into the group 1 (≤ 6.45 mg/g), group 2 (>6.45 and ≤ 10.23 mg/g), and group 3 (>10.23 mg/g), respectively. Non-survivors showed an increased level of BAR at both 1- (*p* < 0.001) and 4-year (*p* < 0.001) follow-up compared with the survivors. The patients with a higher BAR had a higher risk of 1- and 4-year mortality following CABG (33.05 vs. 14.33 vs. 5.14%, *p* < 0.001 and 52.97 vs. 30.72 vs. 13.08%, *p* < 0.001, respectively). Cox proportional hazards regression model suggested a higher BAR as an independent risk factor of 1-year mortality (HR 3.904; 95% CI 2.559–5.956; *P* < 0.001) and 4-year mortality (HR 2.895; 95% CI 2.138–3.921; *P* < 0.001) after adjusting for confounders. Besides, the receiver operating characteristic (ROC) curves showed the better predictive ability of BAR compared to other grading scores at both 1- (0.7383, 95% CI: 0.6966–0.7800) and 4-year mortality (0.7189, 95% CI: 0.6872–0.7506). Subgroup analysis demonstrated no heterogeneous results of BAR in 4-year mortality in particular groups of patient.

**Conclusion:**

This report provided evidence of an independent association between 1- and 4-year mortality after CABG and BAR. A higher BAR was associated with a higher risk of long-term mortality and could serve as a prognostic predictor in patients following CABG.

## Introduction

Coronary artery bypass grafting (CABG) has long been recognized as the most effective myocardial revascularization procedure for patients with advanced coronary artery disease (CAD) (1). This procedure has been performed for more than 40 years to alleviate symptoms and reduce the risk of death in ischemic heart disease (2). While a substantial reported literature on risk assessment following CABG has mainly focused on short- and midterm mortality, few studies have examined predictive indicators for long-term postoperative mortality (3, 4).

Blood urea nitrogen (BUN) is an interesting biomarker that reflects the glomerular filtration rate (GFR) and correlates with postoperative prognosis after cardiac surgery including CABG (4–7). Serum albumin is well-documented for its multiple physiological effects and is widely used during and after cardiac surgery (8). Low-perioperative serum albumin level in patients undergoing cardiac surgery is associated with the increased risk of mortality following surgery and greater incidence of postoperative morbidity, even in the long-term scenario (8–11).

Although both BUN and albumin have been individually applied as predictors of prognosis following cardiac surgery (5, 12, 13), the literature does not contain data investigating the relationship between BAR and long-term mortality after CABG. This retrospective cohort study aimed to explore the role of BAR in predicting long-term mortality in patients following CABG by an analysis of the MIMIC-III database.

## Materials and Methods

### Database Source and Study Population

This retrospective cohort study analyzed the data extracted from the Medical Information Mart for Intensive Care III (MIMIC III) database. This large, publicly available critical care database includes >60,000 patients admitted to the Beth Israel Deaconess Medical Center (BIDMC) from 2001 to 2012 (14). An online training course, Data or Specimens Only Research, was completed by authors to obtain the certification (Record ID: 36309330) for getting access to the database.

A total of 5,411 patients from MIMIC-III database were included who underwent CABG according to ICD-9 code. The exclusion criteria were as follows: (1) patients with more than one ICU admissions (*n* = 404); (2) either BUN or albumin values were absent at admission (*n* = 2,617); (3) patients in the metavision system (*n* = 928). Finally, a total of 1,462 patients who were followed for at least 4-year were included in the study population.

### Data Extraction

The Structure Query Language (SQL) with PostgreSQL (version 9.6) was applied for extracting relevant data from MIMIC-III database including: (1) demographics: age, gender, height, weight; (2) vital signs: heart rate, systolic blood pressure (SBP), diastolic blood pressure (DBP), respiratory rate, temperature and SpO_2_; (3) comorbidities: hypertension, chronic pulmonary disease, diabetes, hyperlipidemia, cerebrovascular disease, chronic liver disease, chronic kidney disease (CKD) and atrial fibrillation (AF); (4) laboratory parameters: BUN, albumin, white blood cell (WBC), hemoglobin, hematocrit, platelet, glucose, creatinine, sodium, potassium and bicarbonate; (5) scoring systems: sequential organ failure assessment (SOFA), acute physiology score III (APS III) and systemic inflammatory response syndrome (SIRS); (6) vasoactive medications: dobutamine, dopamine, epinephrine, norepinephrine, phenylephrine, vasopressin. The laboratory parameters from the first laboratory results were used for analysis. The BAR was calculated by dividing the BUN by the albumin. Postoperative 4-year all-cause mortality was the primary endpoint and 1-year mortality was the secondary endpoint.

### Statistical Analysis

In this study, the optimal cutoff values of BAR for 4-year all-cause mortality were selected with the help of X-tile (version 3.6.1, Yale University School of Medicine, New Haven, Connecticut, USA) software. The Shapiro–Wilk tests were employed for assessing the distribution of variables. Continuous variables were presented as mean ± SD or median and interquartile range (IQR). Categorical variables were presented as numbers and percentages. An ANOVA test or Kruskal–Wallis H-test and the chi-squared test or Fisher's exact test were used to test any significant differences as appropriate. The Kaplan–Meier method with log-rank tests was applied to describe the difference of survival. The univariate and multivariate Cox proportional hazard models were employed for the univariate and multivariate analyses. Variables with a *p* < 0.1 in the univariate model were selected into the multivariable model and the results were presented as hazard ratios (HRs) with 95% CIs. The ROC curves were constructed to evaluate the prognostic efficiency. Subgroup analysis was performed to further verify the role of BAR on the endpoints in subsets of participants using a stratified Cox proportional-hazards regression model. All the statistical analyses were performed using STATA V.14.0, RStudio software (version 1.2.5001), GraphPad Prism 8, and SPSS Statistics 25 (IBM Incorporation, Chicago, Illinois, USA). A two-sided *p* < 0.05 was considered as statistically significant.

## Results

### Patient Characteristics

**Figure 1:**
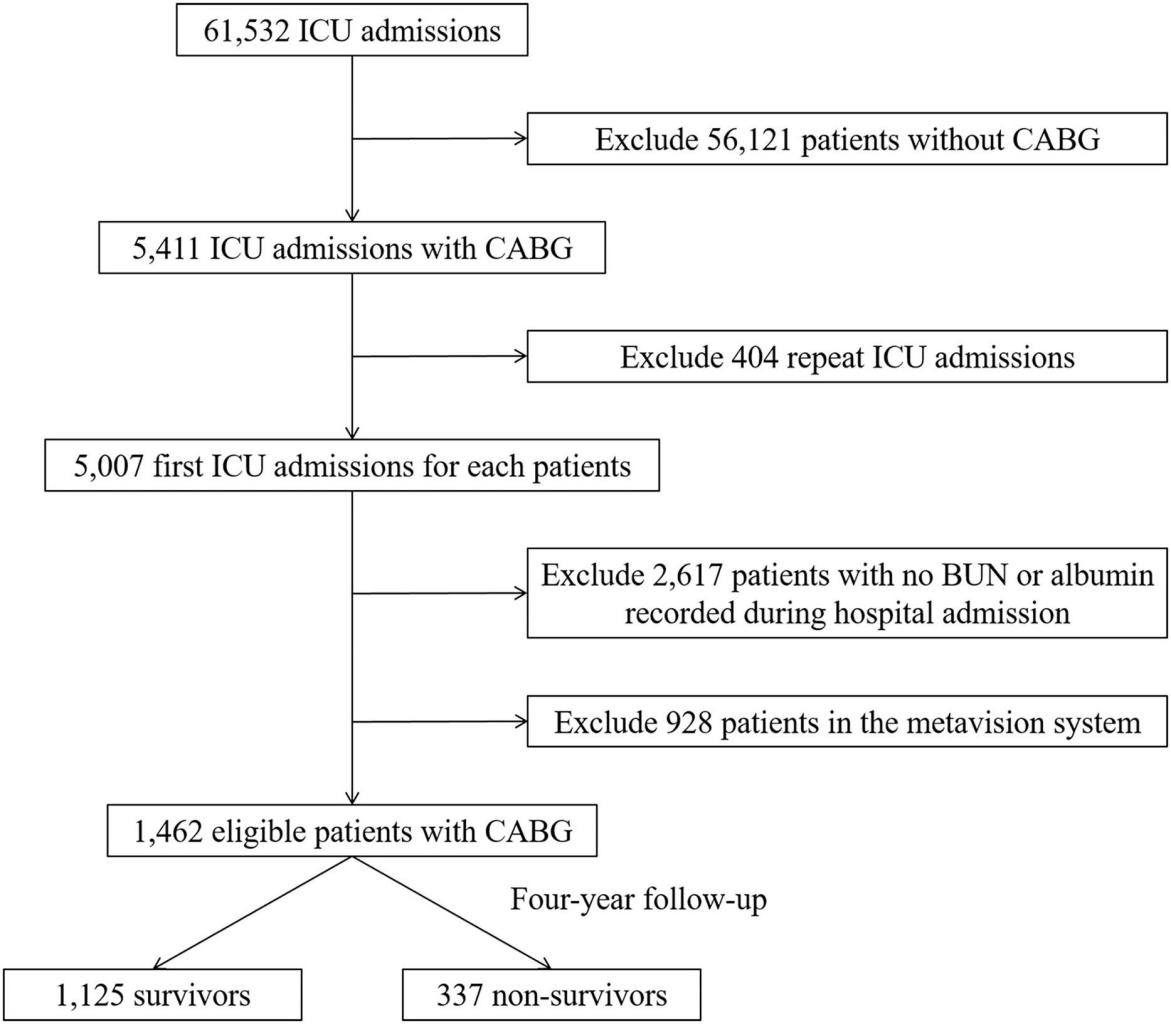
Flow diagram of study patient selection. ICU, intensive care unit; CABG, coronary artery bypass grafting; BUN, blood urea nitrogen.

Initially, 61,532 intensive care unit (ICU) admissions were extracted from MIMIC III database. According to the ICD-9 code, 5,007 patients who underwent CABG with first ICU admission were screened. After excluding the patients with either missing BUN or albumin values (*n* = 2,617) and data from the metavision system (*n* = 928), 1,462 eligible patients were finally enrolled for analysis and categorized into a survived group (*n* = 1,125) and the non-survived group (*n* = 337) after 4-year follow-up. Flow diagram of exclusion and enrollment of study patients is given in Figure 1.

**Figure 2:**
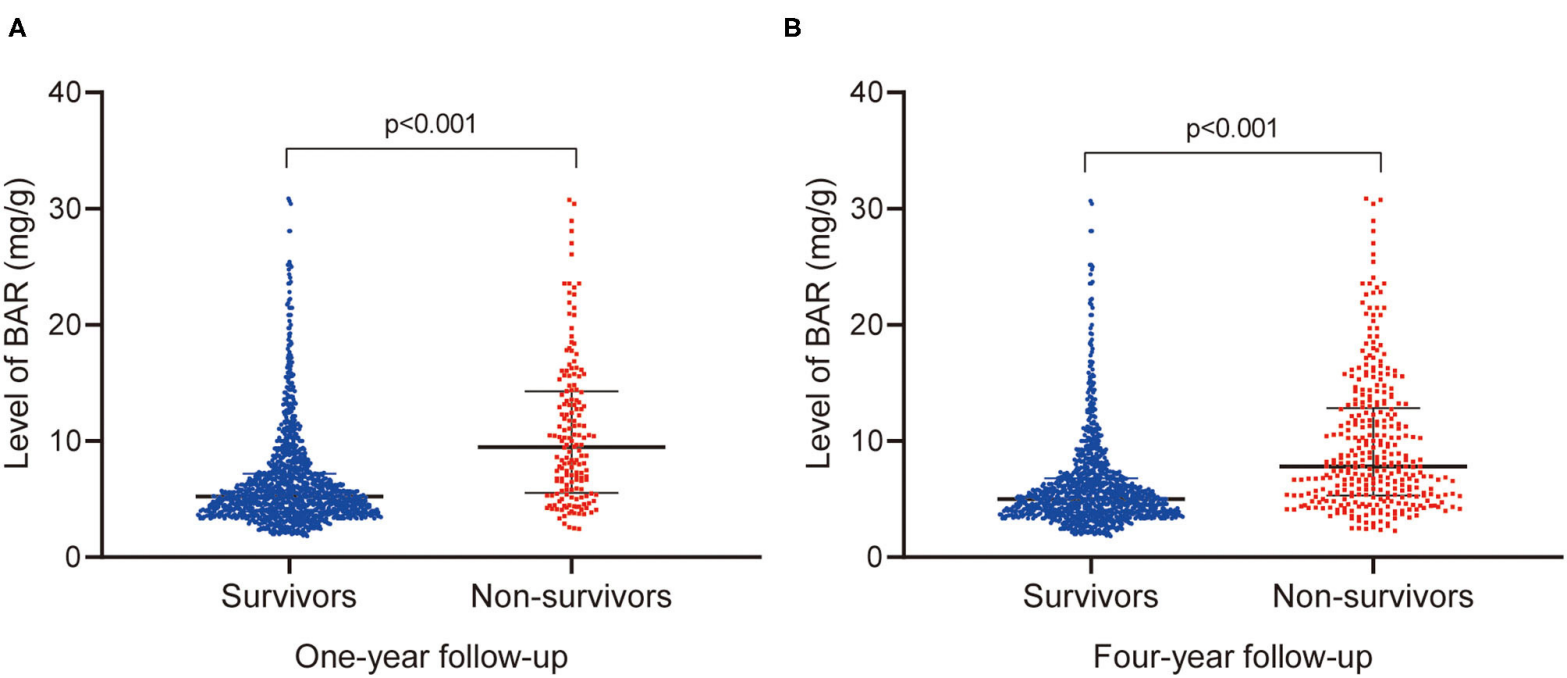
Comparison of BAR levels between survivors and nonsurvivors at 1- **(A)** and 4-year **(B)** follow-up. BAR, blood urea nitrogen-to-albumin ratio.

The patient characteristics of survivors and non-survivors stratified on 4-year mortality were depicted in Table 1. Non-survivors are older compared to survivors (*p* < 0.001). Besides, a higher proportion of chronic pulmonary disease (*p* = 0.009), CKD (*p* < 0.001), AF (*p* < 0.001) and also a higher level of BUN (*p* < 0.001), WBC (*p* = 0.006), creatinine (*p* < 0.001), potassium (*p* = 0.001), SOFA scores (*p* < 0.001), and APS III scores (*p* < 0.001), was observed among non-survivors. The patient characteristics were grouped on 1-year mortality in Supplementary Table 1. Of note, the non-survivors presented an increased level of BAR at both 1-year (*p* < 0.001) and 4-year (*p* < 0.001) follow-up compared with survivors (Figure 2).

**Table 1:** Patient characteristics of survivors and non-survivors at 4-year follow-up.

**Characteristics**	**Survivors (*n* = 1,125)**	**Non-survivors (*n* = 337)**	***p***
Age (years)	69.18 (60.47, 77.10)	76.92 (69.03, 82.65)	<0.001
Male, *n* (%)	808 (71.82%)	212 (62.91%)	0.002
Body mass index (kg/m^2^)	27.66 (25.07, 31.39)	27.02 (23.38, 30.97)	0.001
**Vital signs**			
Heart Rate (beats/minute)	84.95 (78.86, 90.93)	84.95 (78.01, 89.81)	0.664
SBP (mmHg)	112.14 (106.02, 119.36)	112.15 (105.59, 121.29)	0.592
DBP (mmHg)	56.56 (52.98, 61.32)	54.75 (50.41, 59.81)	<0.001
Respiratory Rate (beats/minute)	16.84 (15.20, 19.12)	16.81 (14.86, 19.12)	0.599
SpO_2_ (%)	98.20 (97.23, 98.97)	98.16 (97.18, 99.01)	0.776
**Comorbidities**, ***n*** **(%)**			
Hypertension	725 (64.44%)	149 (44.21%)	<0.001
Chronic pulmonary disease	141(12.53%)	61 (18.10%)	0.009
Diabetes	420 (37.33%)	139 (41.25%)	0.195
Hyperlipidemia	597 (53.07%)	117 (34.72%)	<0.001
Cerebrovascular disease	73 (6.49%)	29 (8.61%)	0.181
Chronic kidney disease	57 (5.07%)	38 (11.28%)	<0.001
Atrial fibrillation	451 (40.09%)	180 (53.41%)	<0.001
**Laboratory parameters**			
BUN (mg/dL)	18.00 (14.00, 24.00)	26.00 (17.00, 38.00)	<0.001
Albumin (g/dL)	3.70 (3.20, 4.00)	3.40 (2.90, 3.70)	<0.001
White blood cell (K/μL)	8.50 (6.80, 11.10)	9.30 (7.10, 12.35)	0.006
Hematocrit (%)	36.40 (32.50, 40.10)	33.70 (30.45, 37.20)	<0.001
Hemoglobin (g/dL)	12.60 (11.30, 14.00)	11.40 (10.40, 12.70)	<0.001
Platelet (K/uL)	213.00 (171.00, 260.00)	210.00 (167.00, 263.50)	0.666
Glucose (mg/dL)	121.00 (100.00, 156.00)	124.00 (103.00, 167.00)	0.188
Creatinine (mg/dL)	1.00 (0.80, 1.20)	1.20 (0.90, 1.70)	<0.001
Sodium (mmol/L)	139.00 (137.00, 141.00)	139.00 (136.00, 141.00)	0.043
Potassium (mmol/L)	4.10 (3.80, 4.40)	4.20 (3.90, 4.60)	0.001
Bicarbonate (mmol/L)	4.20 (3.90, 4.50)	4.20 (3.90, 4.60)	0.031
**Scoring systems**			
SOFA scores	4.00 (3.00, 6.00)	6.00 (3.00, 8.00)	<0.001
APS III scores	34.00 (27.00, 43.00)	44.00 (34.00, 57.00)	<0.001
SIRS scores	3.00 (2.00, 4.00)	3.00 (2.00, 4.00)	0.794
Vasoactive use, *n* (%)	462 (41.07%)	125 (37.09%)	0.192
BAR (mg/g)	5.00 (3.85, 6.79)	7.80 (5.31, 12.83)	<0.001

*SBP, systolic blood pressure; DBP, diastolic blood pressure; BUN, blood urea nitrogen; SOFA, sequential organ failure assessment; APS III, Acute Physiology Score III; SIRS, systemic inflammatory response syndrome; BAR, blood urea nitrogen-to-albumin ratio*.

### Association Between BAR and 1- and 4-Year Mortality After CABG

**Figure 3:**
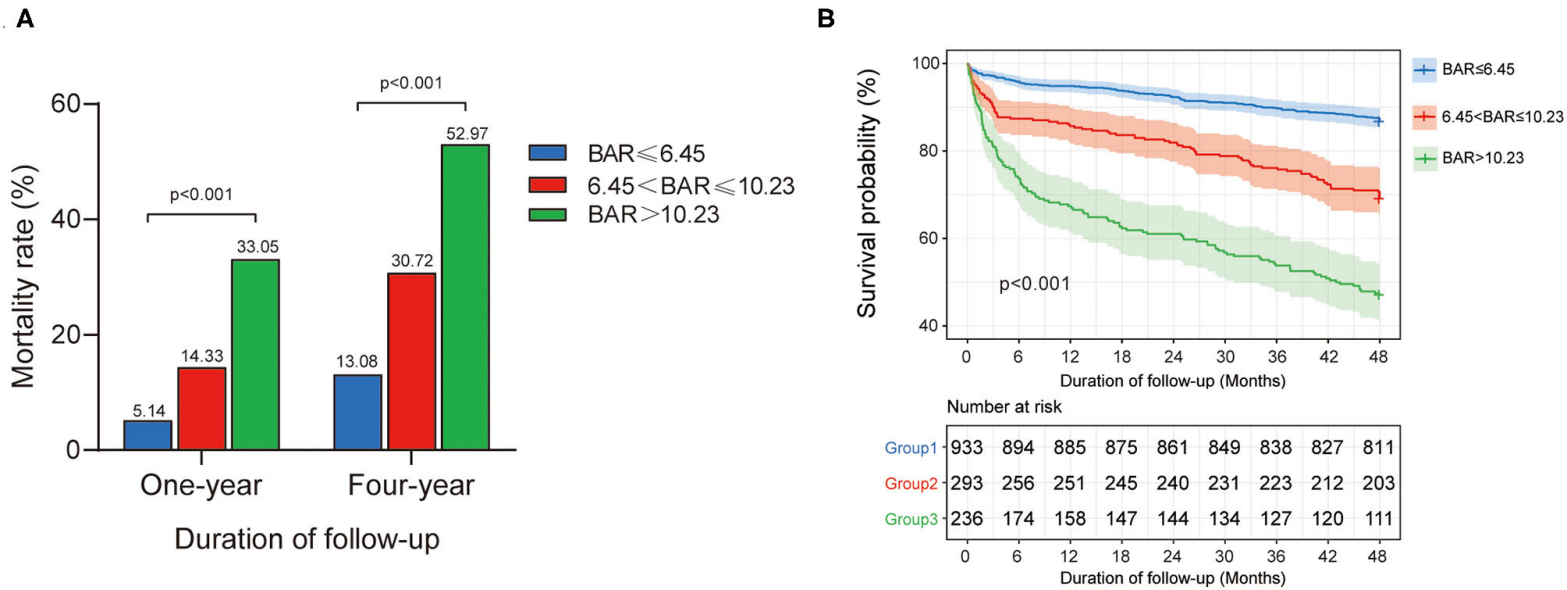
Relationship between BAR and all-cause mortality in patients after CABG: **(A)** the mortality rate in each endpoint according to BAR levels, **(B)** The Kaplan–Meier survival curve of survival probability in patients with different BAR levels. *P*-value was calculated by log-rank test and indicated in the plot. BAR, blood urea nitrogen-to-albumin ratio; CABG, coronary artery bypass grafting.

All the patients were divided into the three groups as group 1 (BAR ≤ 6.45 mg/g, *n* = 933), group 2 (6.45 < BAR ≤ 10.23 mg/g, *n* = 293), and group 3 (BAR > 10.23 mg/g, *n* = 236) according to the cutoff values determined by the X-tile software. The patient characteristics among different groups are given in Table 2. A higher proportion of diabetes, CKD, and AF, along with higher levels of BUN, WBC, glucose, creatinine, potassium, bicarbonate, the SOFA scores, and APS III scores was noticed in group 3. At 1-year follow-up, the mortality in the group 3 was significantly higher compared to the other two groups (33.05 vs. 14.33 vs. 5.14%, *p* < 0.001) (Figure 3A). The result was similar at 4-year follow-up (52.97 vs. 30.72 vs. 13.08%, *p* < 0.001) (Figure 3A). The association between BAR values and postoperative survival was shown with the Kaplan–Meier curves in Figure 3B, indicating that a higher BAR was related to an increased risk of postoperative mortality after CABG (*p* < 0.001).

**Table 2:** Clinical characteristics of patients classified by BAR.

**Characteristics**	**BAR levels (mg/g)**			***p***
	**Group 1: ≤6.45 (*n* = 933)**	**Group 2: > 6.45, ≤10.23 (*n* = 293)**	**Group 3: > 10.23 (*n* = 236)**	
Age (years)	69.13 (60.09, 77.16)	74.66 (66.16, 80.98)	73.80 (64.91, 80.38)	<0.001
Male, *n* (%)	675 (72.35%)	192 (65.53%)	153 (64.83%)	0.017
Body mass index (kg/m^2^)	27.66 (24.87, 31.04)	27.70 (24.63, 31.47)	27.66 (24.54, 32.28)	0.590
**Vital signs**				
Heart Rate (beats/minute)	85.09 (78.94, 91.27)	84.33 (78.73, 89.27)	84.82 (77.33, 90.83)	0.291
SBP (mmHg)	111.92 (105.98, 119.08)	112.93 (105.88, 121.60)	112.14 (105.68, 119.75)	0.328
DBP (mmHg)	56.92 (52.97, 61.57)	55.58 (52.24, 60.43)	54.13 (50.54, 59.22)	<0.001
Respiratory Rate (beats/minute)	16.83 (15.17, 19.06)	17.16 (15.40, 19.44)	16.68 (14.37, 19.02)	0.046
SpO_2_ (%)	98.20 (97.23, 98.98)	98.20 (97.13, 98.92)	98.20 (97.18, 99.11)	0.797
**Comorbidities**, ***n*** **(%)**				
Hypertension	638 (68.38%)	164 (55.97%)	72 (30.51%)	<0.001
Chronic pulmonary disease	127 (13.61%)	42 (14.33%)	33 (13.98%)	0.949
Diabetes	300 (32.15%)	124 (42.32%)	135 (57.20%)	<0.001
Hyperlipidemia	514 (55.09%)	126 (43.00%)	74 (31.36%)	<0.001
Cerebrovascular disease	59 (6.32%)	22 (7.51%)	21 (8.90%)	0.353
Chronic kidney disease	15 (1.61%)	32 (10.92%)	48 (20.34%)	<0.001
Atrial fibrillation	374 (40.09%)	142 (48.46%)	115 (48.73%)	0.007
**Laboratory parameters**				
BUN (mmol/L)	16.00 (13.00, 19.00)	26.00 (23.00, 29.00)	43.00 (35.00, 57.00)	<0.001
Albumin (g/dL)	3.80 (3.40, 4.00)	3.40 (2.95, 3.75)	3.10 (2.50, 3.50)	<0.001
White blood cell (K/μL)	8.40 (6.80, 10.90)	8.80 (6.95, 11.75)	9.40 (7.20, 13.18)	<0.001
Hematocrit (%)	37.10 (33.45, 40.60)	34.10 (31.20, 37.40)	32.40 (29.13, 36.08)	<0.001
Hemoglobin (g/dL)	13.00 (11.60, 14.15)	11.70 (10.70, 12.90)	11.00 (9.90, 12.18)	<0.001
Platelet (K/uL)	216.00 (174.00, 260.00)	204.00 (162.00, 257.00)	208.50 (168.50, 270.50)	0.228
Glucose (mg/dL)	119.00 (99.00, 150.00)	125.00 (105.00, 171.00)	133.00 (103.00, 195.75)	<0.001
Creatinine (mg/dL)	0.90 (0.80, 1.10)	1.20 (1.00, 1.45)	1.90 (1.40, 3.00)	<0.001
Sodium (mmol/L)	139.00 (137.00, 141.00)	139.00 (137.00, 141.00)	138.00 (136.00, 140.00)	<0.001
Potassium (mmol/L)	4.10 (3.80, 4.30)	4.10 (3.90, 4.50)	4.40 (4.00, 4.90)	<0.001
Bicarbonate (mmol/L)	4.10 (3.90, 4.40)	4.20 (3.90, 4.55)	4.40 (3.90, 4.80)	<0.001
**Scoring systems**				
SOFA scores	4.00 (3.00, 6.00)	5.00 (3.00, 7.00)	6.00 (4.00, 8.00)	<0.001
APS III scores	33.00 (25.00, 40.00)	40.00 (33.00, 47.50)	51.00 (41.00, 59.00)	<0.001
SIRS scores	3.00 (2.00, 4.00)	3.00 (2.00, 4.00)	3.00 (2.00, 4.00)	0.828
Vasoactive use, *n* (%)	388 (41.59%)	117 (39.93%)	82 (34.75%)	0.159
**Clinical outcomes**, ***n*** **(%)**				
1-year mortality	48 (5.14%)	42 (14.33%)	78 (33.05%)	<0.001
4-year mortality	122 (13.08%)	90 (30.72%)	125 (52.97%)	<0.001

*BAR, blood urea nitrogen-to-albumin ratio; SBP, systolic blood pressure; DBP, diastolic blood pressure; BUN, blood urea nitrogen; SOFA, sequential organ failure assessment; APS III, Acute Physiology Score III; SIRS, systemic inflammatory response syndrome*.

The Cox proportional hazards regression model was applied to determine the potential independent association between BAR and 1- and 4-year mortality following CABG (Supplementary Table 2 and Table 3). In the univariate model, the risk of 1- and 4-year mortality was higher in the patients with a higher BAR (*p* for trend < 0.001 and < 0.001, respectively). In the multivariate model, the values of BAR remained independently correlated with the risk of 4-year mortality (*p* for trend < 0.001). A consistent result was obtained between BAR and 1-year mortality (*p* for trend < 0.001).

**Table 3:** Cox proportional hazard models exploring the association of BAR with 4-year mortality.

**Variables**	**Univariate model**		**Multivariate model**	
	**Hazard ratio (95% CI)**	***P***	**Hazard ratio (95% CI)**	***P***
**Age (years)**				
≤ 65	Reference	–	Reference	–
>65	2.610 (2.055–3.314)	<0.001	1.999 (1.567–2.551)	<0.001
Male	0.698 (0.560–0.871)	0.001	Not selected	–
Body mass index	0.967 (0.946–0.988)	0.002	0.973 (0.952–0.993)	0.010
**Vital signs**				
Heart Rate	1.001 (0.992–1.011)	0.782	–	–
SBP	1.003 (0.993–1.013)	0.564	–	–
DBP	0.961 (0.946–0.977)	<0.001	Not selected	–
Respiratory Rate	0.991 (0.956–1.027)	0.614	–	–
SPO_2_	0.949 (0.872–1.032)	0.221	–	–
**Comorbidities**				
Hypertension	0.474 (0.382–0.588)	<0.001	0.777 (0.614–0.983)	0.035
Chronic pulmonary disease	1.446 (1.095–1.908)	0.009	1.446 (1.093–1.913)	0.010
Diabetes	1.146 (0.923–1.424)	0.217	–	–
Hyperlipidemia	0.504 (0.403–0.631)	<0.001	0.771 (0.607–0.980)	0.034
Cerebrovascular disease	1.329 (0.908–1.945)	0.143	–	–
Chronic kidney disease	2.062 (1.471–2.890)	<0.001	Not selected	–
Atrial fibrillation	1.596 (1.288–1.977)	<0.001	Not selected	–
**Laboratory parameters**				
BUN	1.035 (1.029–1.040)	<0.001	Not selected	–
Albumin	0.478 (0.407–0.561)	<0.001	0.792 (0.658–0.954)	0.014
White blood cell	1.042 (1.019–1.065)	<0.001	Not selected	–
Hematocrit	0.944 (0.27–0.961)	<0.001	1.049 (1.008–1.092)	0.020
Hemoglobin	0.794 (0.752–0.838)	<0.001	0.809 (0.721–0.909)	<0.001
Platelet	1.000 (0.998–1.001)	0.773	–	–
Glucose	1.002 (1.000–1.004)	0.058	Not selected	–
Creatinine	1.412 (1.317–1.513)	<0.001	Not selected	–
Sodium	0.953 (0.921–0.986)	0.005	Not selected	–
Potassium	1.389 (1.167–1.654)	<0.001	Not selected	–
Bicarbonate	1.345 (1.122–1.613)	0.001	Not selected	–
**Scoring systems**				
SOFA scores	1.151 (1.110–1.194)	<0.001	Not selected	–
APS III scores	1.021 (1.016–1.026)	<0.001	1.010 (1.004–1.016)	0.001
SIRS scores	1.006 (0.901–1.122)	0.918	–	–
Vasoactive use	0.868 (0.696–1.083)	0.210	–	–
**BAR**				
Group 1: ≤ 6.45	Reference	–	Reference	–
Group 2: > 6.45, ≤ 10.23	2.617 (1.993–3.437)	<0.001	1.796 (1.349–2.392)	<0.001
Group 3: > 10.23	5.527 (4.303–7.098)	<0.001	2.895 (2.138–3.921)	<0.001
P for trend		<0.001		<0.001

*BAR, blood urea nitrogen-to-albumin ratio; SBP, systolic blood pressure; DBP, diastolic blood pressure; BUN, blood urea nitrogen; SOFA, sequential organ failure assessment; APS III, Acute Physiology Score III; SIRS, systemic inflammatory response syndrome*.

### Prognostic Efficiency of BAR in 1- and 4-Year Mortality After CABG

**Figure 4:**
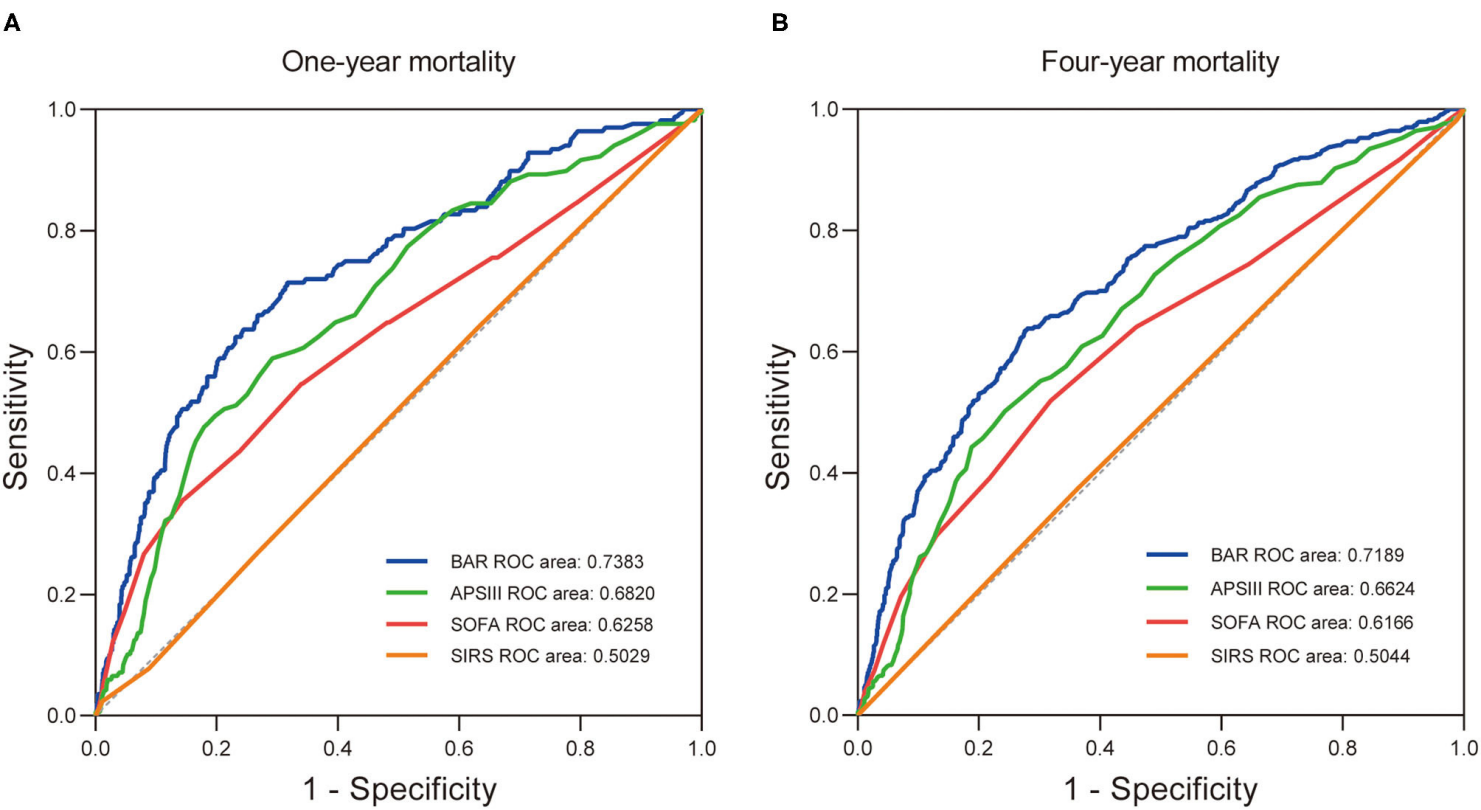
The receiver operating characteristic curves of the predictive value of BAR, APS III, SOFA, and SIRS for 1- **(A)** and 4-year **(B)** all-cause mortality in patients after CABG. BAR, blood urea nitrogen-to-albumin ratio; APS III, acute physiology score III; SOFA, sequential organ failure assessment; SIRS, systemic inflammatory response syndrome; CABG, coronary artery bypass grafting.

**Figure 5:**
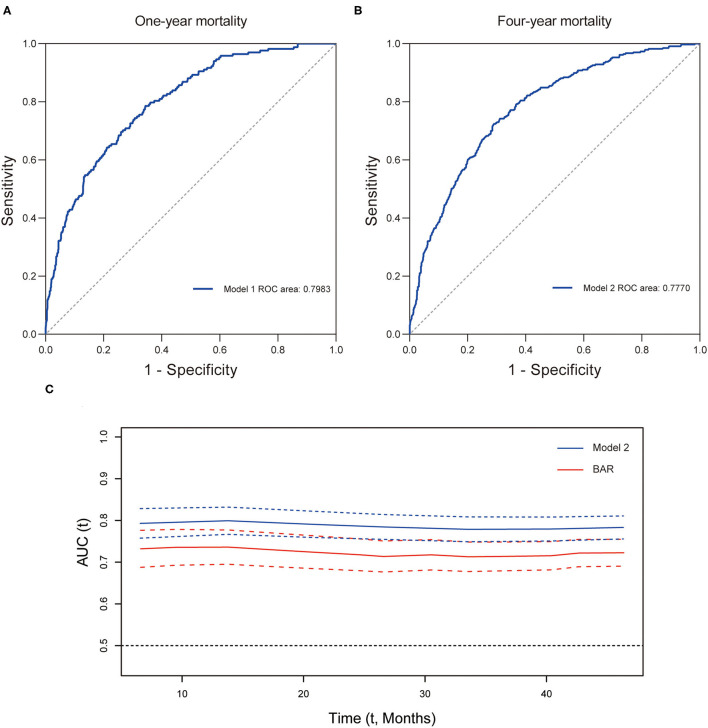
The predictive value of prognostic models for long-term mortality in CABG patients. ROC curve for 1- **(A)** and 4-year **(B)** mortality. **(C)** Time-AUC curves of model 2 and BAR. CABG, coronary artery bypass grafting; ROC, receiver operating characteristic curve; AUC, area under the ROC curve; BAR, blood urea nitrogen-to-albumin ratio.

Furthermore, the prognostic efficiency of BAR and other grading scores (SOFA score, APS III score, and SIRS score) predicting long-term outcomes were compared using ROC curves. For 1-year mortality, the AUC was 0.7383 (95% CI: 0.6966–0.7800) for BAR, 0.6258 (95% CI: 0.5764–0.6751) for SOFA score, 0.6820 (95% CI: 0.6387–0.7253) for APS III score, and 0.5029 (95% CI: 0.4567–0.5490) for SIRS score (Figure 4A). For 4-year mortality, the AUC was 0.7189 (95% CI: 0.6872–0.7506) for BAR, 0.6166 (95% CI: 0.5807–0.6525) for SOFA score, 0.6624 (95% CI: 0.6291–0.6956) for APS III score, and 0.5044 (95% CI: 0.4691–0.5397) for SIRS score, suggesting a better predictive ability of BAR in long-term mortality after CABG (Figure 4B). By incorporating the variables screened out by the multivariate Cox regression, model 1 and model 2 were constructed to predict 1- and 4-year mortality and the ROC curves were constructed to evaluate the prognostic efficiency of BAR and two models. As shown in Figure 5A, the AUC of model 1 for 1-year mortality was 0.7983 (95% CI: 0.7643–0.8323). The AUC of the 4-year mortality of patients with CABG predicted by model 2 was 0.7770 (95% CI: 0.7495–0.8045) (Figure 5B). Besides, the AUCs (95% CIs) of the BAR and model 2 were stable over time (Figure 5C), and the discrimination of outcome was higher for model 2 than for BAR.

### Subgroup Analysis of BAR and 4-Year Mortality After CABG

As shown in Table 4, subgroup analysis was carried out to investigate the heterogeneous results of BAR in 4-year mortality in the particular patient groups. The test for interactions were not statistically significant for age, sex, vasoactive medication, and most comorbidities, including hypertension, chronic pulmonary disease, diabetes, hyperlipidemia, CKD, AF (*p* for interaction = 0.746, 0.108, 0.862, 0.902, 0.557, 0.111, 0.052, 0.351, and 0.316).

**Table 4:** Subgroup analysis of the relationship between BAR and 4-year mortality.

**Characteristics**	**No. of patients**	**BAR levels (mg/g)**			**P for trend**	**P for interaction**
		**≤6.45 HR (95% CI)**	**>6.45, ≤10.23 HR (95%CI)**	**> 10.23 HR (95% CI)**		
Age (years)						0.746
≤ 65	689	1 (ref)	1.682 (0.956–2.959)	2.095 (1.162–3.777)	0.013	
>65	773	1 (ref)	1.828 (1.306–2.560)	3.219 (2.243–4.618)	<0.001	
Gender						0.108
Female	442	1 (ref)	1.925 (1.215–3.051)	2.609 (1.545–4.406)	<0.001	
Male	1,020	1 (ref)	1.680 (1.163–2.425)	2.986 (2.045–4.359)	<0.001	
Hypertension						0.902
Yes	874	1 (ref)	1.556 (1.045–2.316)	2.575 (1.598–4.147)	<0.001	
No	588	1 (ref)	2.108 (1.380–3.220)	3.306 (2.178–5.018)	<0.001	
Chronic pulmonary disease						0.557
Yes	202	1 (ref)	1.341 (0.659–2.731)	1.954 (0.926–4.120)	0.078	
No	1,260	1 (ref)	1.902 (1.382–2.616)	3.231 (2.306–4.527)	<0.001	
Diabetes						0.111
Yes	599	1 (ref)	2.700 (1.625–4.487)	4.265 (2.570–7.075)	<0.001	
No	903	1 (ref)	1.457 (1.016–2.090)	2.334 (1.539–3.541)	<0.001	
Hyperlipidemia						0.052
Yes	714	1 (ref)	2.043 (1.285–3.247)	3.779 (2.264–6.307)	<0.001	
No	748	1 (ref)	1.620 (1.127–2.329)	2.680 (1.841–3.902)	<0.001	
Cerebrovascular disease						0.023
Yes	102	1 (ref)	1.099 (0.402–3.005)	1.248 (0.447–3.487)	0.673	
No	1,360	1 (ref)	1.889 (1.400–2.549)	3.171 (2.306–4.361)	<0.001	
Chronic kidney disease						0.351
Yes	95	1 (ref)	0.960 (0.266–3.460)	1.528 (0.437–5.347)	0.284	
No	1,367	1 (ref)	1.841 (1.367–2.479)	2.984 (2.154–4.132)	<0.001	
Atrial fibrillation						0.316
Yes	631	1 (ref)	1.762 (1.202–2.582)	3.013 (2.002–4.536)	<0.001	
No	831	1 (ref)	1.810 (1.171–2.796)	2.806 (1.785–4.410)	<0.001	
Vasoactive medication						0.862
Yes	587	1 (ref)	1.519 (0.930–2.483)	3.685 (2.160–6.286)	<0.001	
No	875	1 (ref)	1.913 (1.334–2.744)	2.728 (1.863–3.993)	<0.001	

*BAR, blood urea nitrogen-to-albumin ratio; HR, hazard ratio*.

## Discussion

This study for the first time showed that BAR is independently associated with long-term mortality following CABG. Currently, there is no consensus widely accepted regarding a standardized evaluation tool for predicting long-term mortality following coronary revascularization. Establishing a prognostic model including demographical and clinical parameters to predict the risk of long-term mortality after CABG is of importance in the identification of patients at high-risk and timely therapeutic intervention.

Blood urea nitrogen is a blood parameter and its serum level is influenced by renal functions, neurohormonal, and sympathetic activity. BUN has long been recognized to function as an indicator of both cardiorenal dysfunction and neurohormonal activation (15) and a prognostic predictor of long-term mortality in acute and chronic heart failure (HF) (16, 17). Of note, the recent evidence suggested that serum elevation of BUN predicted a worse outcome in patients with acute MI, acute coronary syndrome, and following elective percutaneous coronary procedures (18–20). Recent work reported that BUN-to-left ventricular ejection fraction (LVEF) ratio independently predicted the incidence of long-term major adverse cardiac events (MACEs) including mortality and new-onset decompensated HF in patients undergoing CABG (21). Arnan and his colleagues identified that postoperative BUN was a marker of stroke risk following cardiac surgical procedures (21). Liu et al. observed that BUN could predict the in-hospital mortality of patients with acute aortic dissection (AAD) (7).

Low albumin levels have been considered as a marker of persistent arterial damage and progression of atherosclerosis and thrombosis (22). In the perioperative period, albumin loss, increased capillary permeability, intravenous infusion dilution, and liver dysfunction might be the primary causes of reduced albumin levels in patients (23). Thus, intravenous administration of human-derived albumin is uniformly used in intensive care units and during cardiac surgery (24, 25). Serum albumin maintains the intravascular volume by contributing to the integrity of the vascular wall as well as plasma oncotic pressure (26, 27). Albumin might exert additional benefits of anti-inflammatory and antioxidant effects (28, 29). However, potential adverse effects of albumin use include anaphylactic reactions, prion disease transmission, and acute kidney injury (AKI) (25, 30). A relevant review by Karas and et al. suggested that low-preoperative serum albumin level in patients undergoing cardiac surgery is associated with increased risk of postoperative mortality and morbidity, even in the long-term scenario (9). Beek and his team found that postoperative albumin levels independently correlated with postoperative myocardial damage (10). Engelman et al. reported that hypoalbuminemia independently predicted an increased rate of complications and mortality after cardiac surgery (31). Similarly, another study found that albumin was associated with mortality and morbidity in isolated CABG recipients (32). Kingeter and his colleagues demonstrated that administration of albumin solution was associated with significantly reduced in-hospital mortality and all-cause 30-day readmission rate compared with administration of crystalloids alone in adult on-pump cardiac surgery.

The BAR, a combination of these two parameters, has been reported as a promising indicator of various disease outcomes (33–35). In our study, we calculated the value of BAR based on the preoperative explored the relationship between BAR and the long-term outcomes of patients with CABG and our results aforementioned were consistent with the previous work. Identification of high-risk patients following CABG plays a major role in the prevention and treatment of CAD. Its benefits in clinical assessment might be guiding the prediction of long-term MACEs after CABG and closer follow-up and more active surgical reintervention. With the aid of risk stratification by BAR and also electrocardiogram, echocardiography, coronary angiography, early identification of patients at high risk and timely treatment might be achieved. Besides, the results of the diagnostic test suggested that BAR has a better predictive ability in long-term mortality after CABG. These results are awaiting further verification by the large-scale prospective studies in multiple ethnicities in the future.

### Limitations

There were several limitations that should be highlighted to interpret the results. First, this was a single-center retrospective study based on the MIMIC III public database, and potential selection bias was inevitable. Further studies with large, multicentered, prospective design was necessary to confirm our conclusions. Second, excluding patients with missing values of BUN and albumin might lead to sample selection bias. Third, due to the limited contents of MIMIC III database, some potential risk factors are missing, leading to a certain bias. Fourth, the linearity and proportional hazard assumption for predictor might not be satisfactory in real data, suggesting that the predictive value of BAR needs to be verified by further studies. In addition, machine learning algorithms, which have been widely utilized in the surgical literature, could help address this problem (36, 37). At last, only the results of BUN and albumin for the first time after patient admission were included and their dynamic changes during hospital stay were ignored, which might not precisely reflect the predictive ability of BAR.

## Conclusion

Blood urea nitrogen-to-albumin ratio is independently associated with long-term mortality in patients undergoing CABG. BAR might assist the identification of high-risk patients for closer follow-up and more active surgical reintervention. Future large-scale prospective studies are warranted to verify the results and clarify the underlying mechanisms.

## Data Availability Statement

Publicly available datasets were analyzed in this study. This data can be found here: https://www.physionet.org/content/mimiciii/1.4/.

## Ethics Statement

The studies involving human participants were reviewed and approved by the Massachusetts Institute of Technology (Cambridge, MA) and the Institutional Review Boards of Beth Israel Deaconess Medical Center (Boston, MA). Written informed consent for participation was not required for this study in accordance with the national legislation and the institutional requirements.

## Author Contributions

DZ and XM performed the conception and design of this manuscript. YLiu and ZX provided useful suggestions in methodology. HS, SC, and SZ performed the data analysis. YLi, HZ, and CZ prepared the tables and figures. DZ and XM drafted and revised the manuscript. All authors have read and approved the final manuscript.

## Funding

This study was supported by grants from the National Natural Science Foundation of China (81800255), the National Science Foundation of Shandong Province (ZR2020MH044 to CZ), and the Natural Science Foundation of Shandong Province (ZR2018BH002 to XM).

## Conflict of Interest

The authors declare that the research was conducted in the absence of any commercial or financial relationships that could be construed as a potential conflict of interest.

## Publisher's Note

All claims expressed in this article are solely those of the authors and do not necessarily represent those of their affiliated organizations, or those of the publisher, the editors and the reviewers. Any product that may be evaluated in this article, or claim that may be made by its manufacturer, is not guaranteed or endorsed by the publisher.
