# Supplementary material for: Blood Urea Nitrogen-to-Albumin Ratio in Predicting Long-Term Mortality in Patients Following Coronary Artery Bypass Grafting: An Analysis of the MIMIC-III Database
Source: Front Surg. 2022 Feb 18;9:801708. doi: 10.3389/fsurg.2022.801708 (PMC8894887; doi:10.3389/fsurg.2022.801708)
Supplement: Supplementary file 1 [file Table_1.DOC]

| Characteristics | Survivors (n=1294) | Non-survivors (n=168) | p |
| --- | --- | --- | --- |
| Age (years) | 70.10 (61.39, 78.02) | 76.89 (68.94, 82.97) | <0.001 |
| Male, n (%) | 923 (71.33%) | 97 (57.74%) | <0.001 |
| Body mass index (kg/m^2^) | 27.66 (24.91, 31.40) | 26.70 (23.38, 30.49) | 0.003 |
| Vital signs |  |  |  |
| Heart Rate (beats/minute) | 84.95 (78.59, 90.65) | 85.29 (78.54, 93.23) | 0.159 |
| SBP (mmHg) | 112.14 (106.11, 119.75) | 112.05 (104.43, 120.99) | 0.520 |
| DBP (mmHg) | 56.36 (52.70, 61.05) | 54.98 (50.69, 60.27) | 0.008 |
| Respiratory Rate (beats/minute) | 16.83 (15.19, 19.10) | 16.81 (14.37, 19.45) | 0.653 |
| SpO_2_ (%) | 98.20 (97.24, 98.98) | 98.20 (96.87, 98.92) | 0.367 |
| Comorbidities, n (%) |  |  |  |
| Hypertension | 813 (62.83%) | 61 (36.31%) | <0.001 |
| Chronic pulmonary disease | 176 (13.60%) | 26 (15.48%) | 0.508 |
| Diabetes | 491 (37.94%) | 68 (40.48%) | 0.525 |
| Hyperlipidemia | 667 (51.55%) | 47 (27.98%) | <0.001 |
| Cerebrovascular disease | 84 (6.49%) | 18 (10.71%) | 0.043 |
| Chronic kidney disease | 72 (5.56%) | 23 (13.69%) | <0.001 |
| Atrial fibrillation | 540 (41.73%) | 91 (54.17%) | 0.002 |
| Laboratory parameters |  |  |  |
| BUN (mg/dL) | 18.50 (14.00, 25.00) | 27.00 (17.25, 40.00) | <0.001 |
| Albumin (g/dL) | 3.70 (3.20, 4.00) | 3.20 (2.60, 3.60) | <0.001 |
| White blood cell (K/μL) | 8.50 (6.80, 11.10) | 9.95 (7.50, 13.50) | <0.001 |
| Hematocrit (%) | 35.80 (32.10, 39.70) | 33.60 (29.40, 37.30) | <0.001 |
| Hemoglobin (g/dL) | 12.45 (11.10, 13.80) | 11.30 (10.20, 12.70) | <0.001 |
| Platelet (K/uL) | 214.00 (171.00, 260.25) | 204.00 (165.25, 257.00) | 0.114 |
| Glucose (mg/dL) | 121.00 (101.00, 156.00) | 127.50 (103.00, 171.00) | 0.066 |
| Creatinine (mg/dL) | 1.00 (0.80, 1.30) | 1.30 (1.00, 2.00) | <0.001 |
| Sodium (mmol/L) | 139.00 (137.00, 141.00) | 139.00 (136.00, 141.00) | 0.072 |
| Potassium (mmol/L) | 4.10 (3.80, 4.40) | 4.20 (3.90, 4.60) | 0.003 |
| Bicarbonate (mmol/L) | 4.20 (3.90, 4.50) | 4.30 (3.82, 4.80) | 0.020 |
| Scoring systems |  |  |  |
| SOFA scores | 4.00 (3.00, 6.00) | 6.00 (4.00, 9.00) | <0.001 |
| APS III scores | 35.00 (27.00, 45.00) | 47.00 (35.00, 59.00) | <0.001 |
| SIRS scores | 3.00 (2.00, 4.00) | 3.00 (2.00, 4.00) | 0.898 |
| Vasoactive use, n (%) | 526 (40.65%) | 61 (36.31%) | 0.280 |
| BAR (mg/g) | 5.25 (3.95, 7.18) | 9.48 (5.55, 14.27) | <0.001 |

**Supplementary table 1** Patient characteristics of survivors and non-survivors at one-year follow-up.

SBP: systolic blood pressure; DBP: diastolic blood pressure; BUN: blood urea nitrogen; SOFA: sequential organ failure assessment; APS III: acute physiology score III; SIRS: systemic inflammatory response syndrome; BAR: blood urea nitrogen to albumin ratio.
