# Supplementary material for: Blood Urea Nitrogen-to-Albumin Ratio in Predicting Long-Term Mortality in Patients Following Coronary Artery Bypass Grafting: An Analysis of the MIMIC-III Database
Source: Front Surg. 2022 Feb 18;9:801708. doi: 10.3389/fsurg.2022.801708 (PMC8894887; doi:10.3389/fsurg.2022.801708)
Supplement: Supplementary file 2 [file Table_2.DOC]

| Variables | Univariate model | |  | Multivariate model | |
| --- | --- | --- | --- | --- | --- |
|  | Hazard ratio (95% CI) | P |  | Hazard ratio (95% CI) | P |
| Age (years) |  |  |  |  |  |
| ≤ 65 | Reference | - |  | Reference | - |
| > 65 | 2.626 (1.862-3.704) | <0.001 |  | 1.906 (1.335-2.721) | <0.001 |
| Male | 0.578 (0.425-0.784) | <0.001 |  | Not selected | - |
| Body mass index | 0.956 (0.926-0.986) | 0.005 |  | 0.959 (0.931-0.988) | 0.006 |
| Vital signs |  |  |  |  |  |
| Heart Rate | 1.016 (1.002-1.030) | 0.022 |  | 1.015 (1.001-1.029) | 0.034 |
| SBP | 0.997 (0.983-1.011) | 0.667 |  | - | - |
| DBP | 0.978 (0.957-1.000) | 0.050 |  | Not selected | - |
| Respiratory Rate | 0.998 (0.949-1.049) | 0.941 |  | - | - |
| SpO_2_ | 0.884 (0.788-0.993) | 0.037 |  | 0.874 (0.781-0.977) | 0.017 |
| Comorbidities |  |  |  |  |  |
| Hypertension | 0.360 (0.263-0.493) | <0.001 |  | 0.641 (0.457-0.899) | 0.010 |
| Chronic pulmonary disease | 1.154 (0.759-1.752) | 0.503 |  | - | - |
| Diabetes | 1.096 (0.805-1.491) | 0.561 |  | - | - |
| Hyperlipidemia | 0.386 (0.275-0.540) | <0.001 |  | Not selected | - |
| Cerebrovascular disease | 1.648 (1.010-2.687) | 0.045 |  | Not selected | - |
| Chronic kidney disease | 2.406 (1.550-3.736) | <0.001 |  | Not selected | - |
| Atrial fibrillation | 1.593 (1.176-2.158) | 0.003 |  | Not selected | - |
| Laboratory parameters |  |  |  |  |  |
| BUN | 1.035 (1.027-1.043) | <0.001 |  | Not selected | - |
| Albumin | 0.351 (0.283-0.436) | <0.001 |  | 0.608 (0.478-0.773) | <0.001 |
| White blood cell | 1.068 (1.040-1.097) | <0.001 |  | Not selected | - |
| Hematocrit | 0.938 (0.914-0.963) | <0.001 |  | Not selected | - |
| Hemoglobin | 0.782 (0.724-0.844) | <0.001 |  | Not selected | - |
| Platelet | 0.998 (0.996-1.001) | 0.135 |  | - | - |
| Glucose | 1.003 (1.001-1.005) | 0.011 |  | Not selected | - |
| Creatinine | 1.427 (1.303-1.564) | <0.001 |  | Not selected | - |
| Sodium | 0.950 (0.907-0.996) | 0.033 |  | Not selected | - |
| Potassium | 1.431 (1.129-1.815) | 0.003 |  | Not selected | - |
| Bicarbonate | 1.495 (1.173-1.905) | 0.001 |  | Not selected | - |
| Scoring systems |  |  |  |  |  |
| SOFA scores | 1.189 (1.131-1.250) | <0.001 |  | 1.063 (1.005-1.125) | 0.032 |
| APS III scores | 1.025 (1.018-1.032) | <0.001 |  | Not selected | - |
| SIRS scores | 0.990 (0.849-1.155) | 0.901 |  | - | - |
| Vasoactive use | 0.846 (0.618-1.159) | 0.299 |  | - | - |
| BAR |  |  |  |  |  |
| Group 1: ≤ 6.45 | Reference |  |  | Reference |  |
| Group 2: > 6.45, ≤ 10.23 | 2.947 (1.948-4.459) | <0.001 |  | 1.998 (1.298-3.076) | 0.002 |
| Group 3: > 10.23 | 7.439 (5.190-10.661) | <0.001 |  | 3.904 (2.559-5.956) | <0.001 |
| P for trend |  | <0.001 |  |  | <0.001 |

**Supplementary table 2** Cox proportional hazard models exploring the association of BAR with one-year mortality.

BAR: blood urea nitrogen to albumin ratio; CI: confidence interval; SBP: systolic blood pressure; DBP: diastolic blood pressure; BUN: blood urea nitrogen; SOFA: sequential organ failure assessment; APS III: acute physiology score III; SIRS: systemic inflammatory response syndrome.
